# Drosophila Zinc Finger Protein CG9890 Is Colocalized with Chromatin Modifying and Remodeling Complexes on Gene Promoters and Involved in Transcription Regulation

**DOI:** 10.32607/actanaturae.11056

**Published:** 2020

**Authors:** N. A. Fursova, M. Y. Mazina, J. V. Nikolenko, N. E. Vorobyova, A. N. Krasnov

**Affiliations:** Institute of Gene Biology Russian Academy of Sciences, Moscow, 119334 Russia

**Keywords:** ENY2, CG9890, Drosophila, zinc fingers, ChIP-Seq

## Abstract

In this work, we conducted a genome-wide study of the zinc finger protein CG9890 and showed that it is localized mostly on the promoters of active genes. The CG9890 binding sites are low-nucleosome-density regions and are colocalized with the chromatin modifying and remodeling complexes SAGA and dSWI/SNF, as well as with the ORC replication complex. The CG9890 protein was shown to be involved in the regulation of the expression of some genes on the promoters of which it is located, with the ecdysone cascade genes accounting for a significant percentage of these genes. Thus, the CG9890 protein is a new member of the transcriptional network which is localized on active promoters, interacts with the main transcription and replication complexes, and is involved in the regulation of both basal and inducible transcription.

## INTRODUCTION

Previously, our laboratory isolated and characterized the ENY2 protein that was found to be a component of many of the protein complexes involved in the regulation of transcription and replication. ENY2 is involved in the SAGA, AMEX, and THO transcriptional complexes and connects various stages of gene expression: transcriptional domain organization and chromatin modification, transcription activation and elongation, export of mRNA, and regulation of spatial gene arrangement in the nucleus [1-7]. Also, ENY2 was found to be involved in the ORC replication complex responsible for positioning the replication origin [8-11].

An analysis of the ENY2-Su(Hw) two-hybrid interaction revealed that Su(Hw) recruits the ENY2 protein to the Su(Hw)-dependent insulators of Drosophila, which is necessary for barrier function [5]. Then, Su(Hw) was shown to recruit the histone-acetyltransferase complex SAGA (containing ENY2) [12] and the chromatin remodeling complex dSWI/SNF [13, 14, 15] on Su(Hw)-dependent insulators, causing the formation of a low-nucleosome-density region and creating the conditions for the binding of the ORC replication complex. Knockdown of Su(Hw) almost completely disrupts the recruitment of the SAGA, dSWI/SNF, and ORC complexes to Su(Hw)-dependent insulators and significantly increases the nucleosome density on these regulatory elements [1, 2]. Su(Hw) was shown to be the first example of a protein responsible for positioning the replication origin. Su(Hw) is required for the formation of 6% of the replication origins in the Drosophila genome; therefore, some other, not yet identified, proteins are responsible for the formation of the remaining 94% origins.

Previously, we discovered that there is an interaction between ENY2 and another protein, CG9890, that contains a C2H2-type zinc finger domain, just like Su(Hw) [16]. We reckon that, like Su(Hw), CG9890 is a DNA-binding protein that recruits ENY2-containing complexes to their binding sites, thereby organizing the regulatory genome elements necessary for cell functioning. The CG9890 protein was shown to be localized in the cell nucleus. Biochemical studies revealed an interaction between the CG9890 protein and the ENY2-containing complexes SAGA, ORC, dSWI/ SNF, TFIID, and THOC [16]. CG9890 interacts with the transcriptional complexes involved in transcription initiation and elongation but does not interact with the AMEX complex involved in the export of mRNA from the nucleus to the cytoplasm, which indicates activity of CG9890 at the first stages of the transcription cycle.

In this study, we performed a genome-wide analysis of the CG9890 protein to identify and characterize the regulatory elements for which CG9890 may be responsible.

## EXPERIMENTAL

**Antibodies and cell lines**

In this study, we used the *Drosophila melanogaster *S2 cell line. α-CG9890 polyclonal antibodies were derived from the blood serum of a rabbit immunized with the full-length protein CG9890 expressed in *Escherichia coli *cells [16].

**Chromatin immunoprecipitation and whole genome sequencing**

Chromatin immunoprecipitation was performed according to [1]. For this, we used the CG9890 polyclonal antibodies produced previously [16]. ChIP-Seq libraries were prepared using a NEBNext DNA library preparation kit (New England Biolabs). The quality of the libraries was checked using a Bioanalyzer. For high-throughput sequencing, 200–500 bp fragments were used. The libraries were sequenced on an Illumina HiSeq 2000 genomic sequencer. The produced sequences were mapped to the Drosophila reference genome using the Bowtie2 software. Only uniquely mapped reads were used for further analysis. Identification of the peak coordinates and generation of a full genome profile (WIG file) for the CG9890 protein were performed using the SPP software (FDR < 5%) [17]. A genomic interval of +/–100 bp from the peak position was considered the peak region.

**Bioinformatics analysis**

*D. melanogaster *gene annotations were taken from the official FlyBase website. The genome was divided into the following regions: transcription start sites (TSSs), transcription end sites (TESs), transcribed regions (gene regions except for TSS and TES), and intergenic regions (the others). The ChIP-Seq peak was identified as belonging to one of these categories provided that genomic intervals overlapped at least 10 bp. During peak annotation, the following priority of genomic categories was used: TSS, TES, transcribed and intergenic regions.

## RESULTS AND DISCUSSION

**Protein CG9890 is localized mainly on gene promoters**

**Fig. 1:**
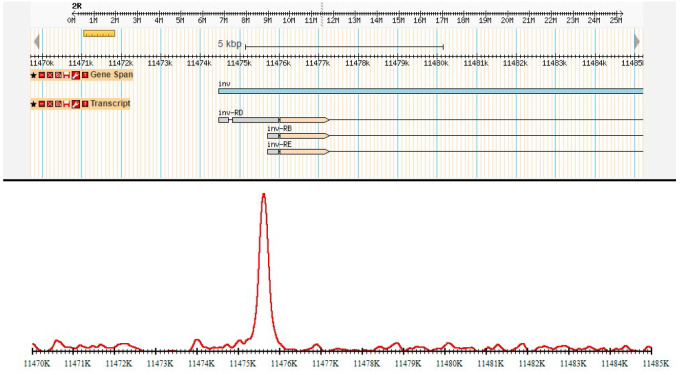
Typical ChIP-Seq profile of the CG9890 protein. The Figure shows a genomic region corresponding to the *Inv *gene promoter. Information about this region from the genome browser is shown on the top panel; the ChIP-Seq profile is shown on the bottom panel

To determine the localization of the studied protein in the genome, we performed chromatin immunoprecipitation from S2 cells using polyclonal antibodies to the CG9890 protein, followed by high-throughput sequencing (ChIP-Seq). A typical ChIP-Seq profile of the CG9890 protein at one of its binding sites is shown in Fig. 1. A total of 4,709 binding sites of the CG9890 protein were identified in the Drosophila genome (FDR < 5%).

**Fig. 2:**
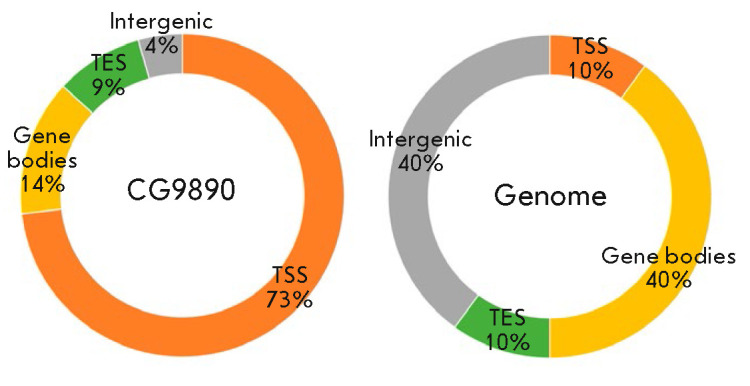
Distribution of the CG9890 protein binding sites relative to the annotated elements of the Drosophila genome (left). For comparison, the relative representation of all annotated elements in the genome is shown (right). TSS – promoter region, TES – end of the gene, Gene bodies – gene region between TSS and TES, Intergenic – intergenic regions

We annotated the identified sites based on their localization in one of the following Drosophila genome elements: promoters, gene ends, gene bodies, and intergenic regions. According to the obtained data (*Fig. 2*), the largest number of ChIP-Seq peaks of the CG9890 protein (73.2%) is localized in the promoter regions of Drosophila genes. We reckon that, being localized predominantly on gene promoters, the CG9890 protein may participate in the functioning of regulatory genetic elements of this type.

**The CG9890 protein is colocalized with chromatin modifying and remodeling complexes in low nucleosome density regions**

**Fig. 3:**
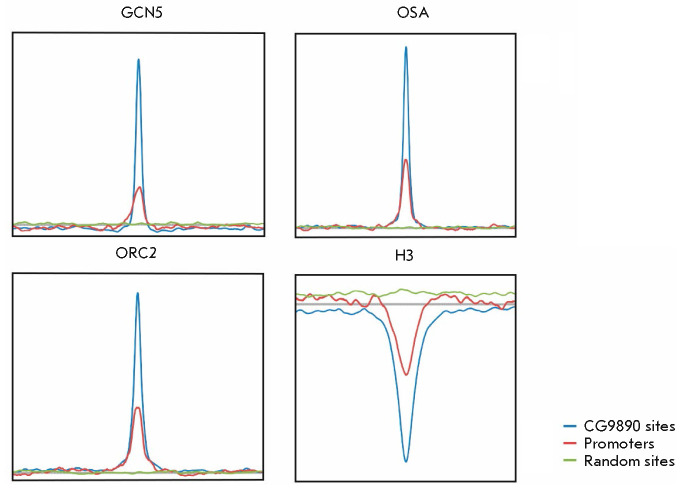
Plots of averaged log2 enrichment ratios for GCN5 (SAGA complex), OSA (dSWI/SNF complex), ORC2 (ORC complex), and Histone H3 at positions –5 to +5 kb relative to the following sites: blue, red, and green plots represent an averaged profile for the indicated factors on the CG9890 sites, randomly selected promoters, and random genome sites (4,709 sites each) in the genome, respectively

Previously, we confirmed the interaction between CG9890 and the ENY2 protein and revealed the interaction between CG9890 and the ENY2-containing protein complexes SAGA, ORC, dSWI/SNF, TFIID, and THO. Therefore, we studied genomic colocalization of the CG9890 protein with the SAGA and ORC complexes, as well as with the dSWI/SNF remodeling complex that, together with the SAGA complex, participates in the formation of the chromatin structure required for the correct functioning of regulatory elements, including promoters. For this purpose, we used software of our own design for generating an averaged profile of the investigated factor at specified genomic sites [1]. The genomic profiles of the ORC2, GCN5, and OSA proteins and histone H3 were previously obtained in our laboratory. Averaged profiles of these proteins were calculated at all 4,709 binding sites of the CG9890 protein, as well as at 4,709 random promoters and 4,709 random genomic sites (*Fig. 3*).

Because the CG9890 protein is located predominantly on gene promoters, there may be enrichment of any promoter factors, including the SAGA, dSWI/ SNF, and ORC complexes, at the CG9890 binding sites relative to the average genome level. However, as seen from *Fig. 3*, the GCN5 (SAGA complex), OSA (dSWI/ SNF complex), and ORC2 (ORC complex) proteins are enriched at the binding sites of the CG9890 protein not only in comparison with the average genome level, but also in comparison with random promoters. This result indicates that this colocalization is associated not just with random coincidence on gene promoters, but with the fact that it is the CG9890 protein binding site that promotes the localization of the SAGA, dSWI/SNF, and ORC complexes. In addition, as follows from *Fig. 3*, the binding sites of the CG9890 protein are characterized by a lower nucleosome density (enrichment of histone H3) than the genome average and on the promoters, which indicates an active state of these regulatory elements.

**Fig. 4:**
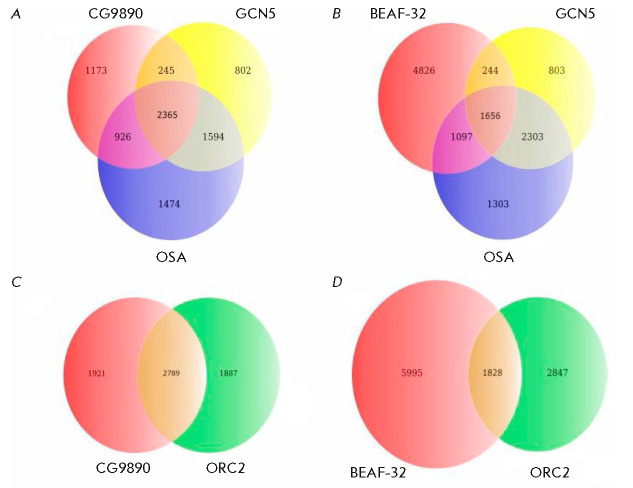
Euler-Venn diagrams showing overlap of the binding sites of the CG9890 and BEAF-32 proteins with the binding sites of GCN5 (SAGA complex), OSA (dSWI/SNF complex), and ORC2 (ORC complex). (*A*) CG9890, GCN5, OSA; (*B*) BEAF-32, GCN5, OSA; (*C*) CG9890 and ORC2; *(D*) BEAF-32 and ORC2

Using the second approach, we calculated the number of CG9890 protein sites overlapping with the GCN5, OSA, and ORC2 protein sites. The well-known protein BEAF-32 was chosen as a control factor [18]. The coordinates of the ChIP-Seq peaks for the BEAF-32 protein were obtained from NCBI GEO (GSE35648). The peaks of two proteins were considered to overlap if their genomic intervals overlapped by at least 10 bp. The obtained data are shown in *Fig. 4*.

As seen from *Fig. 4*, about 60% of the CG9890 protein sites overlap with the sites of the ORC2 protein, a subunit of the ORC complex, which, in turn, accounts for about 60% of the ORC2 protein sites. The level of overlapping of the ORC2 sites with the sites of BEAF-32, another factor localized on the promoters, is significantly lower despite the fact that the number of BEAF-32 protein binding sites in the genome is much higher. *Figure 4* shows that the CG9890 protein is colocalized with the GCN5 and OSA proteins at half of the CG9890 binding sites in the genome, which is significantly higher than an analogous value for the control BEAF-32 protein.

**The CG9890 protein is involved in the regulation of gene expression**

Previously, we demonstrated that the CG9890 protein interacts with the ENY2 protein that coordinates many steps in the regulation of gene expression. The interaction between the CG9890 protein and the ENY2-containing complexes SAGA, ORC, dSWI/ SNF, TFIID, and THOC, i.e. the complexes involved in the initiation and elongation of transcription, was revealed [16]. Given that CG9890 was found predominantly on gene promoters, we decided to investigate, by RNA interference, what changes in the expression of CG9890-associated genes would result from a decrease in the intracellular level of the CG9890 protein. By optimizing the conditions for RNA interference, we achieved an effective decrease in the expression of the studied protein in cells by more than 5 times in terms of the mRNA amount and almost complete depletion of the protein (below the Western blotting detection limit).

**Fig. 5:**
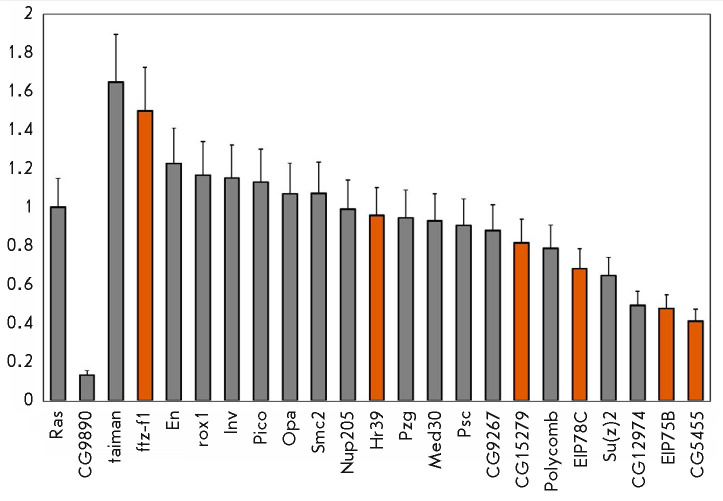
Changes in the expression levels of CG9890-associated genes after RNA interference of the CG9890 protein. The vertical axis shows a change in the mRNA level for the indicated genes after RNA interference relative to the initial level. The *Ras *gene was used for normalization. Error bars correspond to a standard error of the mean. Orange bars correspond to ecdysone cascade genes

By using RT-qPCR, we analyzed the changes in the level of mRNA 21 of the CG9890-associated gene in the cells after RNA interference compared to the control samples. The results of this experiment are shown in *Fig. 5*. After knockdown of the CG9890 protein, the amount of mRNA of seven of these genes decreased by at least 20% and the amount of mRNA in three genes increased by at least 20%. Thus, the CG9890 protein is indeed involved in the regulation of the expression of at least some of the genes on whose promoters it is localized.

Among the 10 genes whose expression changed statistically significantly upon RNA interference of the CG9890 protein, five are ecdysone cascade genes. Their transcription is significantly activated during the response to ecdysone. This enables the use of a convenient model system for cell induction by ecdysone to study in detail the functioning of the CG9890 protein in the regulation of the expression of these genes. The advantage of this system is that it may be used to study the dynamic processes of inducible regulation of gene expression, but not simply to maintain basal transcription [19].

## CONCLUSION

In previous studies, we found that the zinc finger insulator protein Su(Hw) interacts with ENY2 and recruits ENY2-containing complexes to the Su(Hw)-dependent insulators of Drosophila, participating simultaneously in the regulation of transcription and the positioning of replication origins. We also established an interaction between ENY2 and another protein, CG9890, containing a zinc finger domain, like Su(Hw). Biochemical studies revealed an interaction between the CG9890 protein and the ENY2-containing complexes SAGA, ORC, dSWI/SNF, TFIID, and THOC. We suggest that, like Su(Hw), the CG9890 protein is a DNA-binding protein that recruits ENY2-containing complexes to their binding sites, thereby organizing the genome regulatory elements necessary for cell functioning. In this study, we identified the CG9890 binding sites in the genome and showed that they are located mainly on gene promoters. We found a genome-wide correlation between CG9890 binding sites and the ENY2-containing complexes SAGA, ORC, and dSWI/SNF. The CG9890 protein binding sites are characterized by a lower nucleosome density (enrichment of histone H3) than the genome and promoter averages, which indicates an active state of these regulatory elements. The CG9890 protein is involved in the regulation of the expression of some genes on the promoters of which it occurs, with the ecdysone cascade genes accounting for a significant percentage of these genes. Thus, the CG9890 protein is a new member of the cell transcriptional network which is localized on active promoters, interacts with the main transcription and replication complexes, and is involved in the regulation of both basal and inducible transcription.

## Abbreviations

ENY2: enhancer of yellow 2
C2H2: zinc fingers of C2H2 type
SAGA: histone acetyltransferase complex
SWI/SNF: chromatin remodeler
AMEX: mRNA export complex
ORC: origin recognition complex
